# Spectral Tuning of a Nanoparticle-on-Mirror System by Graphene Doping and Gap Control with Nitric Acid

**DOI:** 10.1021/acsami.3c05302

**Published:** 2023-08-03

**Authors:** Julia Lawless, Oisín McCormack, Joshua Pepper, Niall McEvoy, A. Louise Bradley

**Affiliations:** † School of Physics and AMBER, Trinity College Dublin, College Green, Dublin 2, Ireland; ‡ School of Chemistry and AMBER, Trinity College Dublin, College Green, Dublin 2, Ireland

**Keywords:** nanophotonics, plasmon coupling, nanoparticle-on-mirror, graphene, 2D materials

## Abstract

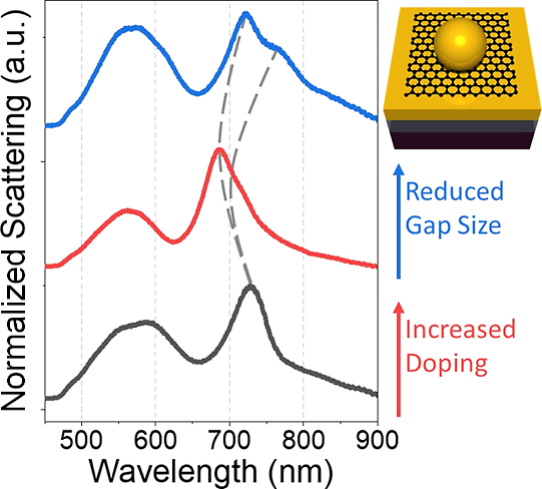

Nanoparticle-on-mirror systems are a stable, robust, and reproducible method of squeezing light into sub-nanometer volumes. Graphene is a particularly interesting material to use as a spacer in such systems as it is the thinnest possible 2D material and can be doped both chemically and electrically to modulate the plasmonic modes. We investigate a simple nanoparticle-on-mirror system, consisting of a Au nanosphere on top of an Au mirror, separated by a monolayer of graphene. With this system, we demonstrate, with both experiments and numerical simulations, how the doping of the graphene and the control of the gap size can be controlled to tune the plasmonic response of the coupled nanosphere using nitric acid. The coupling of the Au nanosphere and Au thin film reveals multipolar modes which can be tuned by adjusting the gap size or doping an intermediate graphene monolayer. At high doping levels, the interaction between the charge-transfer plasmon and gap plasmon leads to splitting of the plasmon energies. The study provides evidence for the unification of theories proposed by previous works investigating similar systems.

## 1 Introduction

The precise control of the separation between two metal nanoparticles is of high interest due to the many applications involved exploiting the ability to trap light at the sub-wavelength scale.^1,2^ These include optical switching,^3,4^ energy harvesting,^5^ and molecular detection and sensing.^6−8^ The use of these nanogaps has made it possible to greatly increase the confinement of light to scales of less than 1 nm.^1^ It is possible to achieve this effect by confining the light between two nanoparticles, for example, in a bowtie formation. This can be done by creating an array of nanoparticles by electron beam lithography, allowing the precise positioning of the nanoparticles to give control over the nanogaps created.^9−11^ Utilizing a nanoparticle-on-mirror (NPoM) approach, however, gives stronger and more reproducible gap control. The NPoM approach retains the same confinement effects as with two nanoparticles coupled together but involves the coupling of a nanoparticle with its mirror image.^1,12,13^

It is interesting to combine two-dimensional (2D) materials with the NPoM geometry. This is because the light is confined within dimensions similar to the material of atomic thickness. This gives rise to a pathway for much stronger light–matter interaction for 2D materials, allowing much deeper exploration into their optical properties.^14−18^ Another advantage of utilizing 2D materials in these systems is that their thicknesses are well known and constant at the monolayer limit. This constant gap thickness can be useful in keeping the gap size fixed while monitoring the other changes in the plasmonic system.^19^

A well-explored 2D material is graphene, the thinnest of the 2D materials, with a thickness of 0.34 nm.^16,20,21^ Graphene has many unique properties, including its high electrical conductivity and its high chemical stability.^22−25^ Graphene has a tunable dielectric response that can be modified by doping both chemically and electrically. This can be manipulated for a wide variety of applications, including hyperlenses, metacouplers, photovoltaic devices, and super-resolution imaging and sensing.^18,26,27^ Many techniques have been developed to allow for the growth of a large surface area and a high-quality monolayer film, such as chemical vapor deposition, flame synthesis, and pulsed laser deposition.^28^ Many studies have explored the coupling of graphene to plasmonic structures, mostly in the infrared region. It has been demonstrated that graphene can be used to modulate the plasmonic response by modifying the dielectric properties.^29−37^ This can be achieved by both electrical gating and chemical doping. When the graphene layer overlaps with the electric field hot spot, it can have remarkable effects on the modulation of the plasmonic spectra, even in the visible wavelength range.^16,20,34,38^

Two separate groups, Shao et al.^20^ and Mertens et al.,^16^ investigated a system involving an Au nanosphere on top of an Au film, with an intermediate layer of graphene. These systems demonstrated optical modulations, tuned by graphene, in the visible to near-infrared spectrum. This makes these works particularly interesting as most systems involving the tuning of plasmonic energies with graphene are limited to the infrared region. Both works showed the formation of three different peaks in the scattering spectra. Shao et al. attributed the three peaks to the octupolar, quadrupolar, and dipolar modes. Mertens et al. attributed the three peaks to the transverse plasmon and the coupling between the charge-transfer plasmon (P_CTP_) and gap plasmon (P_GAP_).

In this paper, we present evidence to show that both of these theories are correct, and we demonstrate the conditions under which each is the dominant effect. Our system involves a Au nanosphere of 150 nm in diameter, on top of a 100 nm thick Au film, with an intermediate layer of graphene. The Au film is on top of a 100 nm thick SiO_2_ layer on an Si substrate. Several plasmon modes are visible in the scattering spectrum of this system. Nitric acid is employed to modulate these modes by two different mechanisms. It serves to dope the graphene and also to reduce the gap size in between the Au nanosphere and film. Our results demonstrate the unification of the theories presented by both Shao et al. and Mertens et al. and the regimes in which each is observed.

## 2 Materials and Methods

The monolayer graphene was grown by chemical vapor deposition (CVD) on commercial polycrystalline copper foils using methane as the hydrocarbon precursor, similar to the technique employed by Li et al.^25^ This method was chosen due to its high monolayer coverage. The CVD graphene was transferred onto the target substrates using a polymer-mediated etching and transfer process,^25,39,40^ utilizing poly(methyl methacrylate) (PMMA) as the handling polymer and ammonium persulfate as the copper etchant. The PMMA coating was removed by heating to 150 °C and washing in acetone. The gold layer was deposited on the SiO_2_/Si substrates by electron beam evaporation with a Temescal. The Au nanospheres were synthesized with a wet chemistry approach using the method of Zheng et al.^41^ This method was chosen to obtain a high yield of nanospheres that were uniform in size and highly spherical.

A SEM image of a sample of synthesized nanospheres is shown in Figure S1. The product was diluted in ethanol and sonicated for ≈3 min before drop-casting onto the substrates. The 150 nm Au nanospheres were drop-cast onto the Au film with a graphene monolayer on top, the Au film without a graphene monolayer, the SiO_2_/Si substrate with a graphene monolayer on top, and the SiO_2_/Si substrate without a graphene monolayer. The substrates were heated for 10 min in acetone at 40 °C after the deposition to reduce the CTAB (cetrimonium bromide), [(C1_6_H_33_)N(CH_3_)_3_]Br layer surrounding the particles.^32^ This also helped to reduce the amount of residual PMMA remaining on the graphene. The measurements of scattering spectra were taken at the single particle level under white light excitation with a dark field microscope using a 100× objective lens of 0.9 numerical aperture. The spectra were recorded with an Andor CCD camera and an Andor 230i spectrometer. Approximately 50 measurements were taken for single nanospheres drop-cast onto each of the four different substrates considered.

FDTD simulations were carried out using the commercial software, Lumerical solutions. These were used to illustrate how the gap size and changes to the permittivity of the graphene layer modulate the energy of the plasmon modes. The nanospheres were modeled with a diameter of 150 nm and approximated as perfect spheres. The dielectric function of Au was taken from a fit of the points measured by Johnson and Christy.^42^ A thickness of 0.5 nm was used for the graphene layer, which was modeled according to Stauber et al.^43^ This model was chosen as it is valid for visible wavelengths. The doping of the graphene layer was modeled by changing the chemical potential energy (Fermi level) from 0.1 to 1.2 eV. The change of the Fermi level shifts the electron valence band level in the Dirac cone-approximated band structure of graphene.^44^ The gap between the nanosphere and graphene layer was modeled with a permittivity of 1.5 to approximate any residual polymer and the CTAB layer.^45^

**Figure 1:**
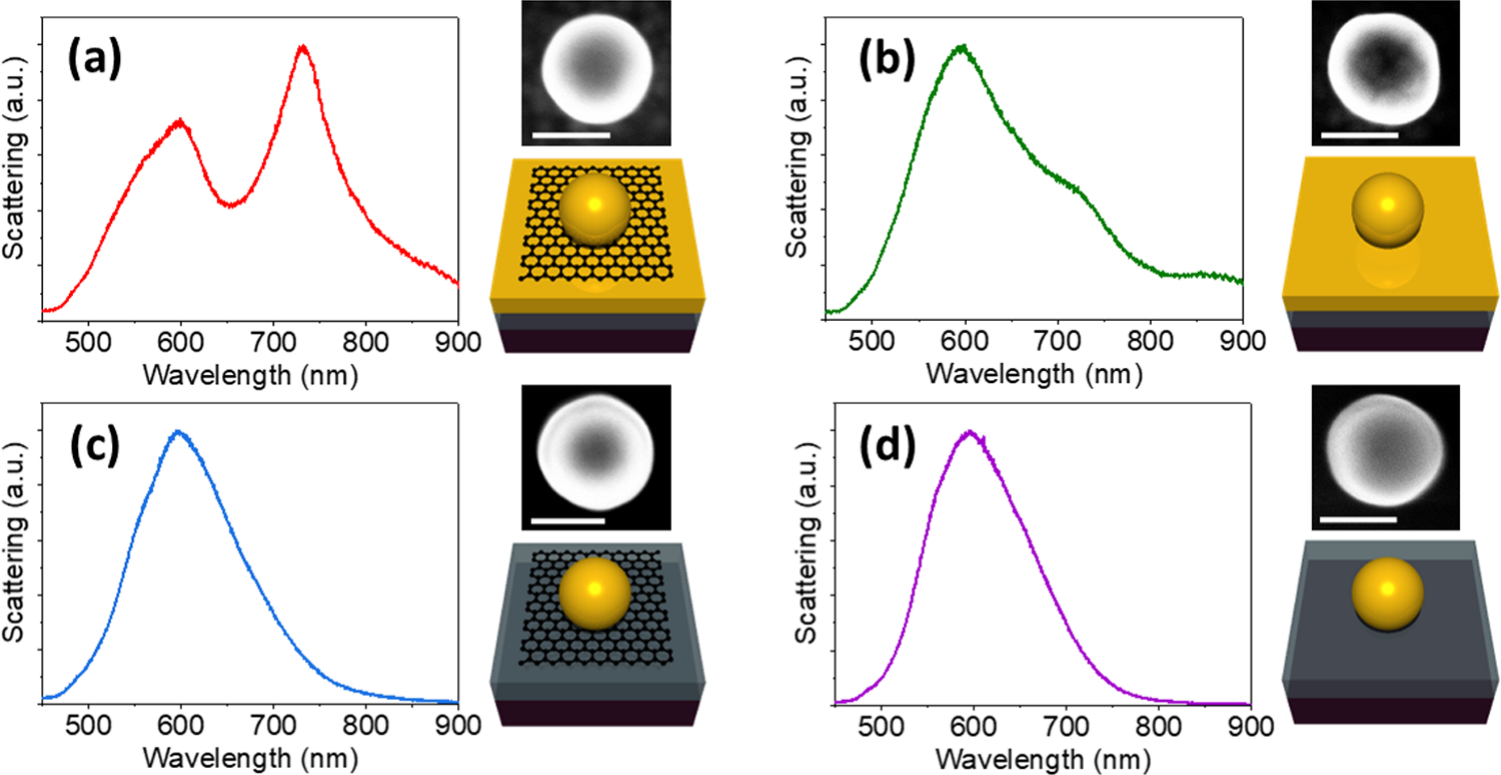
(a–d) Experimental scattering results from single 150 nm Au nanospheres (a) on top of a monolayer of undoped graphene on a Au film, (b) directly on top of the Au film, (c) on top of a monolayer of undoped graphene directly on the SiO_2_/Si substrate, and (d) directly on the SiO_2_/Si substrate. A SEM image of the particle measured, with scale bar showing 100 nm, is shown at the upper right of each plot. A schematic of each system is shown at the lower right of each plot underneath the sphere.

**Figure 2:**
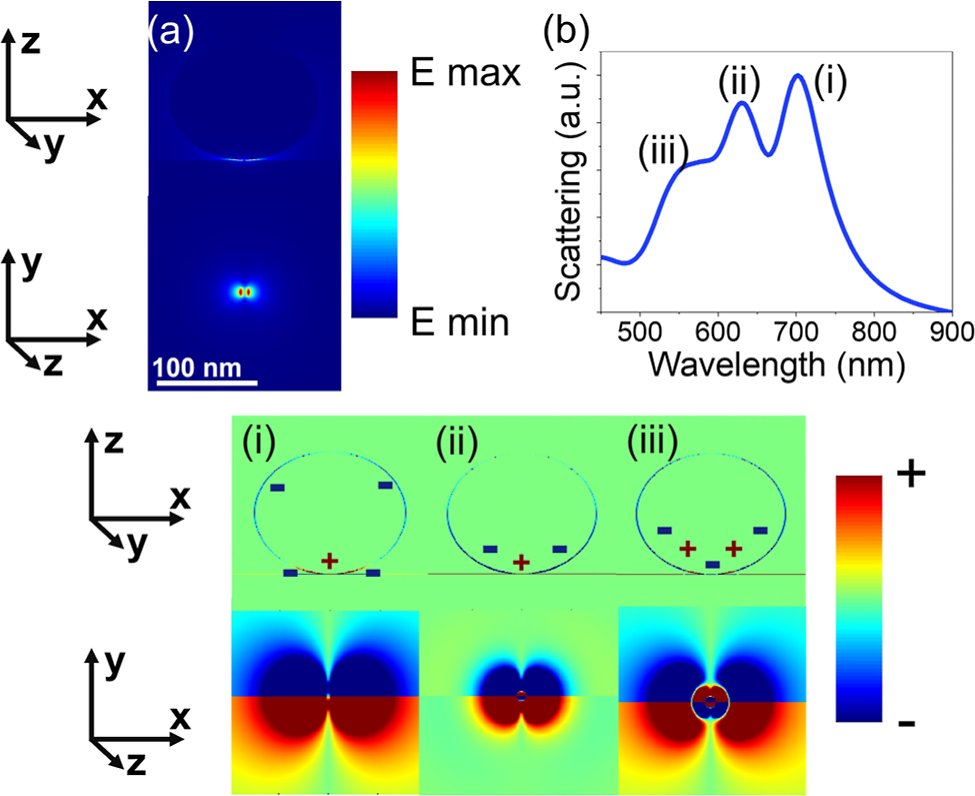
Simulated results from a 150 nm Au nanosphere on a monolayer of graphene on the Au film. The gap between the Au sphere and graphene layer is 1 nm, and the graphene has a chemical potential of 0.1 eV. (a) Electric field map at the energy of the quadrupolar mode. (b) The simulated scattering spectrum is also shown with the dipole, quadrupole, and octupole peaks labeled (i), (ii), and (iii), respectively. The corresponding charge distribution maps at the energies of the dipole, quadrupole, and octupole are also shown. The cross-section of each map is 200 × 200 nm.

Example experimental scattering spectra of four different nanospheres are shown in Figure 1a–d. Corresponding SEM images of the particles measured are shown to the right of each scattering spectrum, and a schematic of each system is shown underneath. The scattering spectrum of the Au nanosphere and Au film with the intermediate monolayer of graphene is shown in Figure 1a. Three peaks are visible in this spectrum, corresponding to the in-plane octupolar (570 nm), quadrupolar (597 nm), and dipolar (732 nm) modes, as identified by Shao et al.^20^ The octupolar peak is visible as a shoulder to the quadrupolar peak. The electric field map from a simulation of this system is shown in Figure 2a at the energy corresponding to the quadrupolar mode. (The distribution of the electric field strength was found to be the same for all multipolar modes studied. The quadrupolar mode is shown here because it had the largest electric field strength within the gap region when compared with the dipolar and octupolar modes.) The light in this simulation was approximated as s-polarized, coming from directly above the nanosphere. The top panel shows the cross section through the center of the sphere and the substrate underneath, and the bottom panel shows the cross section on the top of the Au film directly underneath the sphere and graphene. It is clear from these two maps that the electric field is highly confined in the gap between the Au sphere and film, where the graphene is positioned. This elucidates how this structure can be utilized to maximize the interaction of the plasmonic resonances with a graphene monolayer. The simulated charge distribution maps are shown in Figure 2i–iii. These maps show the same planes through the center of the sphere and the substrate and at the top of the Au plane, underneath the nanosphere, as in the electric field map in Figure 2a. They are recorded at the energy of each of multipolar modes, as shown in Figure 2b, and with a chemical potential of 0.1 eV. The lowest energy peak, corresponding to the peak at 732 nm in the experimental scattering spectrum in Figure 1a, is clearly a dipolar mode. Similarly, higher orders of multipoles are evident in Figure 2b, corresponding to the peaks at 597 and 570 nm in the experimental spectrum. The top panel in Figure 2i shows some features that do not correspond to the dipolar mode. This is because some of the charge in the Au sphere leaked into the polymer layer. Simplified results of the same simulation with the polymer area replaced with air are shown in Figure S2, demonstrating a clearer dipolar mode when there is no polymer layer for the charge to leak into.

An experimental scattering spectrum of the same system, without the graphene monolayer, is shown in Figure 1b. This spectrum also shows several peaks corresponding to different multipolar modes. There is some variation of where the peaks were positioned for this system, across the different particles measured. As will be discussed below, this is due to the high sensitivity of the plasmon modes to the gap size between the Au nanosphere and film. This gap can vary by up to ≈1 nm between nanoparticles. This gap is due to the CTAB layer coating the nanospheres, residual from after heating the substrates in acetone. All four substrates were heated in 40 °C acetone to reduce the CTAB layer after the nanospheres were drop-cast. Experimental scattering spectra of Au nanospheres directly on the SiO_2_/Si substrate, with and without a monolayer of graphene, are shown in Figure 1c,d, respectively. Both spectra show one broad peak at ≈600 nm, composed of the different multipolar modes that are too close together to resolve. The lack of the Au film underneath significantly reduces the electric field strength and the interaction between the plasmons and the graphene, resulting in no notable differences between the spectra of the spheres with and without a monolayer of graphene between them and the SiO_2_/Si substrate.

## 3 Effect of Graphene Doping and Gap Size

**Figure 3:**
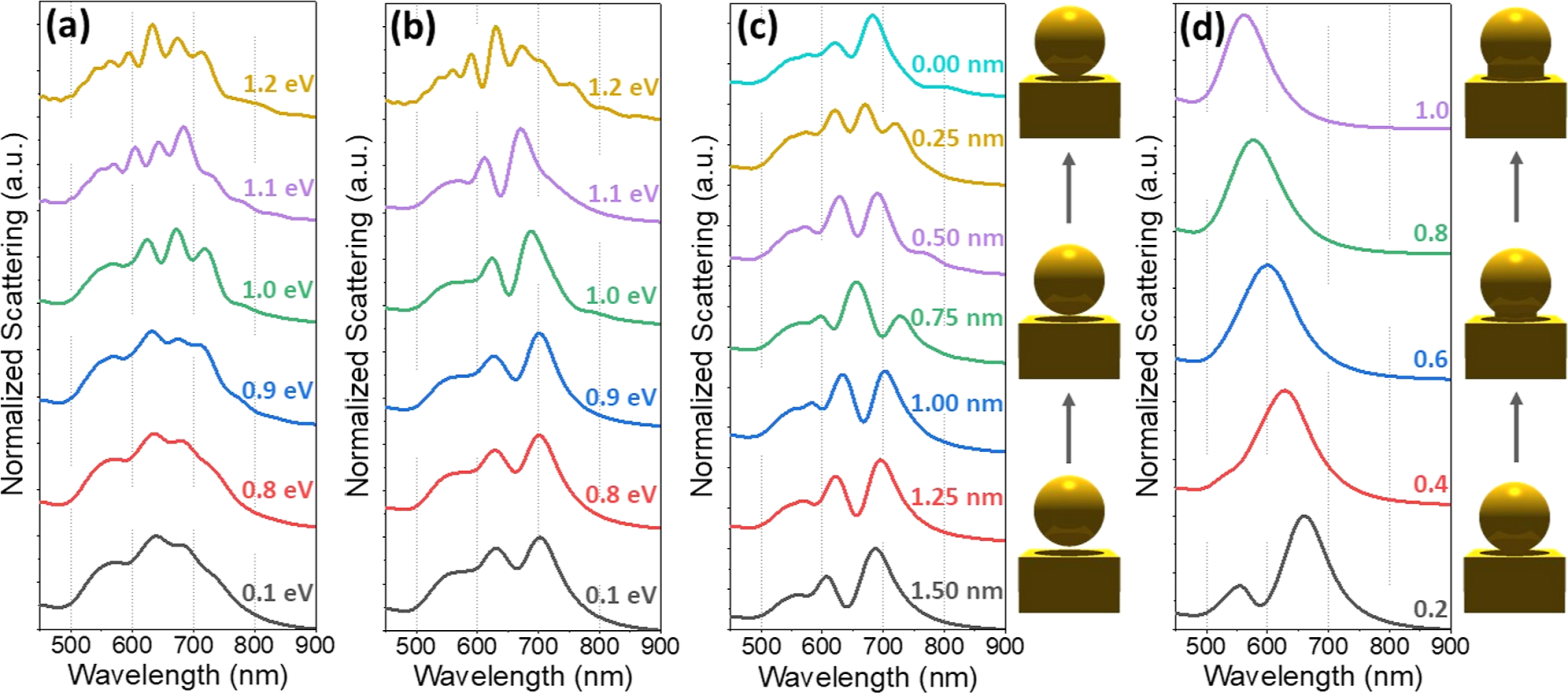
(a,b) Simulated scattering spectra for a 150 nm Au sphere on a 100 nm Au film, sandwiching a monolayer of graphene on a SiO_2_/Si substrate with a 0.25 and a 1 nm gap between the sphere and the graphene, respectively. The chemical potential of the graphene is increased from 0.1 to 1.2 eV. (c) Simulated scattering spectra for the same system, without the graphene layer. The gap between the Au film and Au sphere is reduced from 1.5 to 0 nm. This effect is illustrated to the right. (d) Simulated scattering of the same system as in (c), but with no gap between the Au sphere and film. The system is simulated with a neck formed between the sphere and film, with the ratio of the neck to sphere diameter increased from 0.2 to 1. This effect is illustrated to the right.

Gap plasmons have a very high sensitivity to their environment. Therefore, a myriad of factors can cause slight discrepancies between the experimental results from different particles, including the particle shape, size, gap size, and surface roughness of the Au film. Due to these small differences between different particles, the simulations employed in this paper are used only to show global trends. The systems corresponding to the spectra in Figure 1 are simulated with an idealized model, assuming that the nanoparticles are perfect spheres of 150 nm diameters and are placed on substrates with completely flat surfaces. These approximations are used to keep the simulations simple and to best observe the trends involved in the evolution of the spectra as the gap size between the nanosphere is modified or as the graphene layer is doped. This simple model is not used to find an exact fit for each experimental spectrum. Therefore, there is a slight discrepancy between the peaks corresponding to each mode in the experimental spectra (shown in Figure 1) and the simulated spectra (shown in Figure 3).

The effect of the doping of the graphene layer is shown in Figure 3a,b. Both plots show the simulated scattering spectra of an Au nanosphere on top of a 100 nm Au film, separated by a monolayer of doped graphene. In Figure 3a, the gap between the sphere and the top of the graphene layer is 0.25 nm, while in Figure 3b, the gap is 1 nm. The total gap between the Au sphere and film is the gap between the Au sphere and the graphene, plus the thickness of the graphene layer. In both cases, the dipolar mode is shown to blue-shift as the doping level goes up. This effect is more extreme in the case of the smaller gap size. It blue-shifts by ≈45 nm in Figure 3a when the doping is increased from 0.1 to 1.1 eV, but it only blue-shifts by ≈30 nm in Figure 3b. This is due to the increased electric field confinement and enhancement overlapping with the graphene layer when there is a smaller gap. The doping of the graphene layer, therefore, has a larger effect on the system when the gap is as small as possible. The blue-shifting of the graphene is due to the reduction of the real part of the permittivity of the graphene at higher doping levels.^6,20,30^ When the doping level is as high as 1.2 eV, the dipolar mode splits in two in Figure 3b, and all of the modes split in Figure 3a. The mechanics behind this phenomenon will be discussed in more detail below.

Another difference between the spectra in Figure 3a,b is seen in the higher energies of the modes in the latter. This is more evident when looking at the sharper peaks when the graphene is more highly doped. For example, when the chemical potential is set to 1 eV, the dipole mode is positioned at 688 and 717 nm when the gap size is 1 and 0.25 nm, respectively. This effect can be understood by comparing the system to a capacitor, with the charge built up on either side of the gap, driven by the incoming light. This higher energy is due to the increased gap, causing a reduced capacitance between the Au sphere and film, increasing the oscillation period. This effect is also seen in Figure 3c, where the system is modeled without a graphene layer. The gap between the nanosphere and film is reduced from 1.5 to 0 nm, with the edge of the sphere merely coming into contact with the film. The scattering peaks are seen to red-shift,^46^ with more multipolar modes appearing as the gap size is brought to just 0.25 nm. A schematic illustrating the effect of the sphere being brought closer to the gold film is shown to the right of this plot. The PMMA layer modeled as a medium with a refractive index of 1.5 has the effect of some extra small peaks forming in the spectra due to the charge leaking described in Section 2. Versions of Figure 3a–c are shown in Figure S3, with the PMMA modeled as air instead. These plots give a clearer view of the trends occurring due to the change of gap size and chemical potential.

**Figure 4:**
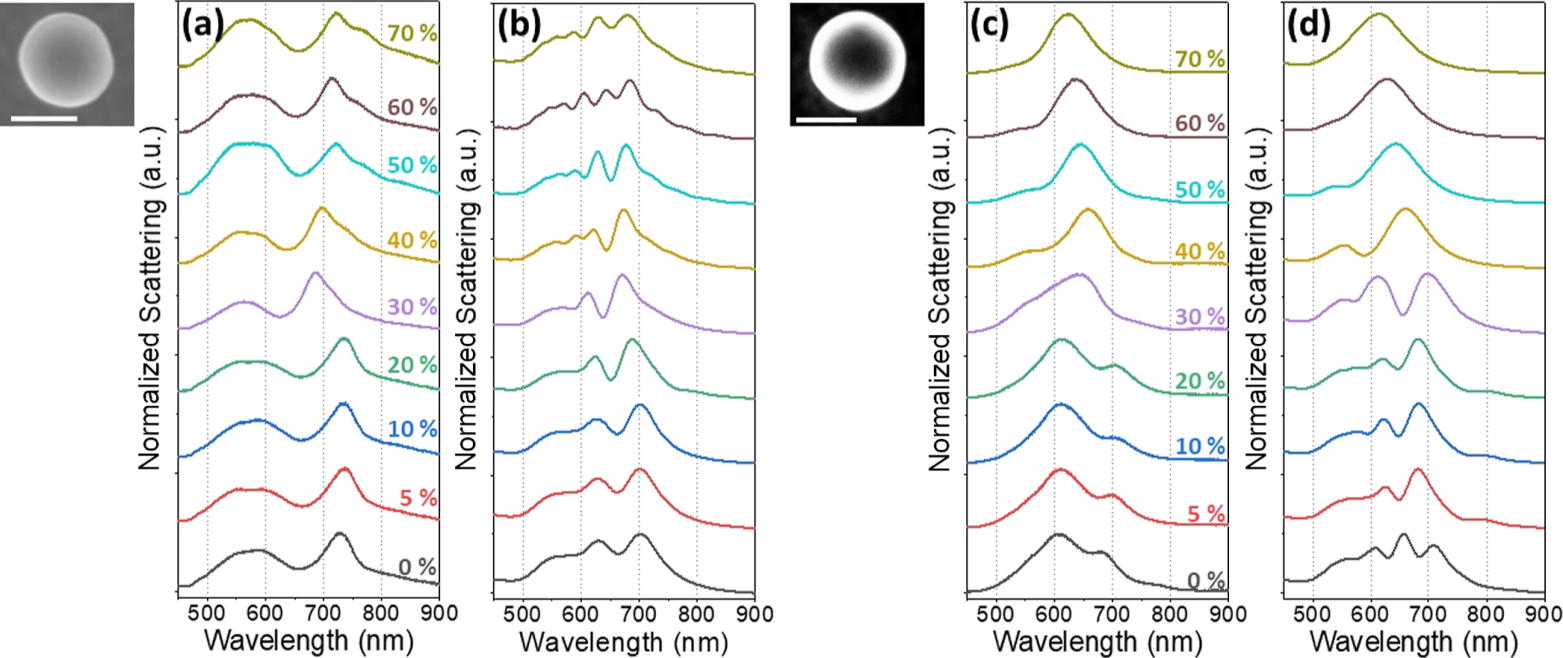
(a,b) Experimental and simulated scattering spectra of the same nanosphere after being immersed for 5 min in nitric acid of increasing concentrations. The 150 nm Au nanosphere is on top of a monolayer of graphene on a 100 nm Au film. An SEM image of the nanosphere is shown to the left, with the white scale bar showing 100 nm. (c,d) Experimental and simulated scattering spectra of the same nanosphere after being immersed for 5 min in nitric acid of increasing concentrations. The 150 nm Au nanosphere is directly on top of the 100 nm Au film, with no intermediate layer of graphene. A SEM image of the nanosphere is shown to the left, with the white scale bar showing 100 nm.

Another feature evident in Figure 3c is the reduction of the intensity of the dipolar mode when the Au sphere and film are brought <1 nm apart. This effect is also observed in the experimental spectra in Figures 4c and S6 and the simulated spectra in Figure 4d. The cause for this reduction of intensity is beyond the scope of this paper and warrants further investigation.

When a gold nanoparticle is brought into contact with a gold substrate, a neck is formed between them by the rearrangement of the atoms of the nanoparticle touching the sphere.^16,47−49^ With the formation of this neck, it is no longer possible for the multipolar modes to form because the capacitor effect cannot occur without the gap region between the Au sphere and film. Simulations of this effect are shown in Figure 3d, with the radius of the neck being increased from 20% of the radius of the nanosphere to 100% of the radius of the nanosphere. This is illustrated to the right of the plot. When the neck is still small compared to the radius of the diameter, some plasmonic modes are still visible in the scattering spectrum, suggesting that the sphere can still couple with the film for very small neck sizes. As the neck size is increased, however, the scattering peaks collapse into one, showing that the coupling between the sphere and film is no longer happening. As the neck radius increases, the plasmon is shown to blue-shift. This result is supported by similar findings in the literature.^47,49^

## 4 Control of Graphene Doping and Gap Size with Nitric Acid

The two effects described above were experimentally realized by the immersion of the samples in nitric acid (HNO_3_). The nitric acid was used as a tool to effectively p-dope the graphene layer and also etch away the residual CTAB and PMMA between the Au sphere and film, reducing the gap size between the two. The samples were immersed in 5, 10, 20, 30, 40, 50, 60, and 70% nitric acid for 5 min each. The nitric acid had the effect of p-doping the graphene.^50^ Raman spectra were taken of the graphene after each immersion in various concentrations of nitric acid. The results are shown in Figure S4 and can confirm a high level of doping across the entire graphene monolayer.^50,51^ The doping is estimated to be between 0.9 and 1.1 eV after being immersed in 70% nitric acid, with slight variations across the graphene sample. This is evidenced by the Raman spectra presented in Figure S4(52) and the experimental spectra shown in Figures 4a and S5.

Dark-field scattering spectra were obtained in between each immersion of the sample in nitric acid for all 50 nanospheres measured on each substrate type. The results of the sample spectra are shown in Figure 4. As seen in Figure 3, the plasmon modes are highly sensitive to both the gap size and the doping of the graphene layer. Therefore, there was a variety seen in the change of the spectra for the different particles measured. Despite these differences, the overall trends remain constant across all of the experimental spectra.

An example of a 150 nm Au nanosphere on a 100 nm Au film, separated by a monolayer of graphene, is shown in Figure 4a, with experimentally measured dark-field spectra for each concentration of nitric acid up to 70%. Further examples of experimental spectra for each concentration of nitric acid are shown in Figure S5. As for the simulated results shown in Figure 3, the largest changes occurred for the dipolar mode. The dipolar mode is seen to red-shift slightly as the nitric acid concentration is brought from 0 to 20%. This is expected, because before the sample is immersed in a high concentration of nitric acid, plenty of residual CTAB and PMMA is still present, causing a large gap between the Au sphere and film. Therefore, as seen in Figure 3, as the graphene is doped, only a slight blue-shift would be expected. Further, as demonstrated in Figure 3a,b, blue-shifts due to the doping of the graphene only occur at high chemical potentials, demonstrating why a blue-shift is only apparent when the 30% nitric acid is used. The other effect of the nitric acid is the etching away of the material in the gap region, reducing the size of the gap. This has the effect of red-shifting the plasmon energy. Both effects together are shown by a very slight red-shift of about 6 nm. A significant blue-shift of 50 nm is shown when the sample is immersed in 30% nitric acid. This indicates that the graphene is now heavily doped, possibly reaching the chemical potential of about 1 eV. As the nitric acid concentration is increased further, the plasmon energy is shown to red-shift slightly again, indicating that the gap is still reducing in size. Another interesting effect is that the dipolar mode has begun to split into two peaks, as shown in Figure 3. This indicates that the chemical potential has been brought up to about 1.1 eV.

The same system was simulated, as shown in Figure 4b, with the chemical potential being increased from 0.1 to 1.1 eV and the gap size being reduced from 1 to 0 nm. The simulations show the same trend as in Figure 4a, with the doping level and gap size chosen to match as close as possible with those in the experiments. The doping level was increased from 0.1 to 1.1 eV in the first five spectra shown, matching the trend in the experiments from 0 to 30% nitric acid. The gap size was then reduced from 1 to 0 nm for the remaining spectra, matching the 30 to 70% nitric acid spectra in the experiments. There is a slight discrepancy in the energy of the dipolar mode, as the simulations predicted it to have a higher energy. The other difference between the simulations and experiments is that the gap change and doping level appear to have a larger effect on the quadrupolar mode in the simulations than they do in the experimental data.

Figure 4c shows an example of another Au sphere on a 100 nm Au film, this time without the monolayer of graphene in between. Further examples of experimental spectra for each concentration of nitric acid are shown in Figure S6. The plasmon energies are again seen to shift due to the change in the gap region. The peaks red-shift slightly as the nitric acid concentration is increased to 30%. The positions of the peaks indicate that the gap size is very small before the sample is immersed in nitric acid, especially the dipolar peak at ≈780 nm. The dipolar peak is very diffuse with a low intensity when there is a small gap, as demonstrated in Figure 3c. The reason for this very small gap is because the sample was immersed in acetone heated to 45 °C before the measurements were taken. This removed a lot of the CTAB around the particles. The other reason is that there was no PMMA coating the Au film as there was coating in the graphene, which helped to keep the gap small. As the nitric acid concentration is increased to 70%, the multipolar modes disappear, giving rise to one single plasmon mode, as shown in Figure 3d. This is a clear indication that a neck is formed between the Au sphere and film, aided by the immersion in the nitric acid. The plasmon is shown to blue-shift with each immersion from 40 to 70%, indicating that the neck is increasing in diameter as the nitric acid concentration increases. This could be due to the nitric acid removing more CTAB with each immersion, freeing more Au atoms on the lower side of the nanosphere to rearrange and fuse with the Au film underneath.

The scattering spectra for this system were also approximated with simulations. As the nitric acid concentration was increased to 20%, simulations were carried out with a gap size reduced from 0.3 to 0 nm. As the nitric acid concentration was increased further to 70%, the neck radius was increased from 0 to 50% of the radius of the sphere’s diameter. It can be seen in both the experimental and simulated results that as the gap decreases, the plasmons red-shift. After the sphere comes into contact with the Au film, the plasmons start to blue-shift again as the neck increases in diameter. This blue-shift is due to a reduced capacitance as the neck becomes more substantial, allowing more charge to tunnel through. The experiment and simulation show very similar results as the nitric acid concentration reaches 40%, giving good predictions as to how thick the neck becomes after the treatment with nitric acid. Most of the nanospheres measured showed evidence of a neck being formed with a diameter of 40 to 60% of the diameter of the nanosphere.

Experimental scattering spectra for a 150 nm nanosphere on a SiO_2_/Si substrate, with and without a graphene layer in between, immersed in 0 to 70% nitric acid, as in Figure 4, are shown in Figure S7. No change in these spectra are detected after treating the samples with nitric acid. This confirms that the shifts observed with the Au film underneath are only possible when the electric field is confined in the small volume between the Au sphere and film.

## 5 Increasing the Coupling Strength with a Small Gap

**Figure 5:**
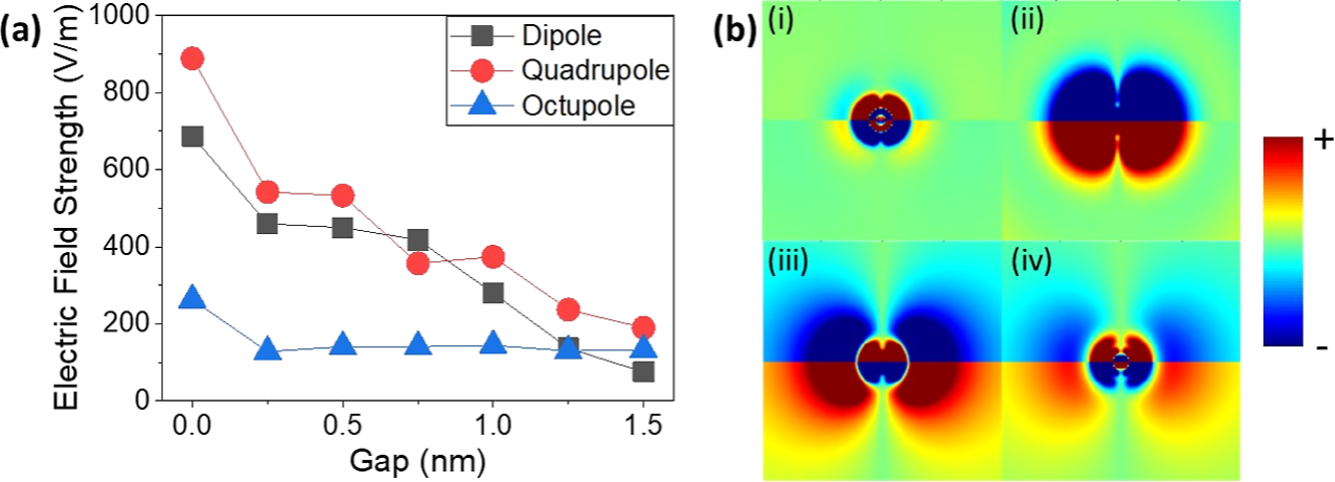
Simulated results for a 150 nm Au nanosphere on a 100 nm Au film separated by a monolayer of graphene. (a) Plot showing the maximum electric field strength within the graphene layer at the energy of the dipolar, quadrupolar, and octupolar modes for varying gap sizes between the Au sphere and graphene layer. The chemical potential is fixed at 0.1 eV. (b) Charge distribution maps at the surface of the Au film, directly underneath the nanosphere. The gap between the sphere and graphene layer is 1 nm, and the graphene is doped with a chemical potential of 1.2 eV, as shown in Figure 3b. The maps correspond to the position of the peaks at (i) 673 nm, (ii) 629 nm, (iii) 590 nm, and (iv) 558 nm. The cross section of each map is 200 nm × 200 nm.

We have seen that the doping of graphene results in a blue-shift of the plasmon modes, but the shift is larger for smaller gap sizes. This is clear when comparing Figure 3a,b. The reason for this can be found by examining the electric field strength within the graphene layer. The maximum electric field strength within the graphene layer is shown in Figure 5a. The electric field strength is calculated at the energy of the dipolar, quadrupolar, and octupolar modes for a 150 nm Au sphere on an Au film, sandwiching a graphene monolayer with a chemical potential of 0.1 eV. The gap between the Au sphere and the graphene layer was varied from 0 to 1.5 nm. For each of the multipolar modes, the E field is clearly shown to reduce as the gap was made larger. This shows the importance of keeping the gap size small and minimizing the thickness of the CTAB and PMMA layers. It also elucidates how this system can be utilized with fine control to achieve very high electric field intensities, concentrated in the graphene layer. The electric field strength is shown to reach as high as 885 V/m for the dipolar mode when there is no gap between the nanosphere and graphene layer. This is a significant field enhancement as the incident field strength in Lumerical solutions is 1 V/m. The electric field strength is further increased with a higher chemical potential in the graphene layer, with an approximately two-fold increase when the chemical potential is raised from 0.1 to 1.1 eV. This indicates that the graphene layer has a stronger influence on the plasmonic modes at higher doping levels, also evidenced by the splitting of the dipolar mode.

We have seen that the peaks in the scattering spectra of the NPoM system correspond to the multipolar modes as described in Shao et al.^20^ However, we have not yet examined the theory given by Mertens et al.,^16^ theorizing that the two lower energy peaks are due to the charge-transfer plasmon (P_CTP_) and the gap plasmon (P_GAP_) interacting. The charge-transfer plasmon is the dipolar resonance of the whole system. The gap is conductive and gives rise to an equal and opposite charge in the Au sphere and the Au mirror film, as demonstrated in Figure 2. The gap plasmon is highly localized in the vicinity of the gap and is therefore more dependent on the doping of the graphene layer. The two plasmons interact as follows
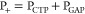
1
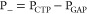
2

As the doping gets larger, P_GAP_ increases, causing the dipolar mode to split into two new modes, P_+_ and P_–_. This is supported by both experimental results and simulations (see Figures 3 and 4). For smaller gaps, the quadrupolar and octupolar modes are also seen to split by the same mechanism in the simulated spectra. This is seen by the additional narrower peaks which appear when the chemical potential of the graphene is particularly high or the gap size is particularly low (Figure 3a,b). This demonstrates that a much stronger interaction with the graphene is required for the quadrupolar and octupolar modes to split than is required for the dipolar mode.

Further simulations were carried out to ensure that the charge distribution matched this theory. A 150 nm sphere on an Au film, separated by a monolayer of graphene with a chemical potential of 1.2 eV, was simulated with a gap of 1 nm between the sphere and graphene layer as in Figure 3b. The charge distribution was investigated at the wavelengths of the peaks shown in the scattering spectrum, at 673, 629, 590, and 558 nm. The results are shown in Figure 5b. The peaks at (iii) 590 and (iv) 558 nm show the quadrupolar and octupolar modes, as expected, matching the maps shown in Figure 1. Interestingly, the peak at (i) 673 nm also appears octupolar, despite being lower in energy compared to the peak at (ii) 629 nm, which is clearly dipolar. These results indicate that the dipolar plasmon has split into two new plasmons, P_+_ and P_–_. P_–_ occurs as a result of P_CTP_ and P_GAP_ acting in opposite directions. This has the effect of reducing the energy of the plasmon and also distorting the charge distribution. This gives it the appearance of an octupolar mode, despite being lower in energy. P_+_ occurs as a result of P_CTP_ and P_GAP_ acting in the same direction. This has the effect of enhancing the dipolar effect and also increasing the plasmon energy.

## 6 Conclusions

We have investigated a single Au nanoparticle on an Au mirror film separated by a monolayer of graphene. We have demonstrated with both numerical simulations and experimental results how sensitive the plasmon modes are to both the gap size and the level of doping in the graphene layer, even at visible wavelengths. The coupling of the nanosphere and film gives rise to multipolar modes which can be tuned to blue-shift by doping a monolayer of graphene in between them or by altering the gap size. At high doping levels, the plasmons are seen to split into two new energy levels. Our results serve to explain how different theories in the two previous papers by Shao et al. and Mertens et al. are both correct under different levels of doping and different gap sizes. These results will be useful for the design and fabrication of future devices employing the NPoM system separated by a 2D material. They give a deeper understanding of the interactions between the different plasmonic modes and how they can be controlled.

## Supporting Information Available

SEM image of synthesized Au nanospheres, additional charge distribution maps, additional simulated and experimental scattering spectra, and Raman maps of areas of a graphene sample (PDF)
