# Supplementary material for: Spectral Tuning of a Nanoparticle-on-Mirror System by Graphene Doping and Gap Control with Nitric Acid
Source: ACS Appl Mater Interfaces. 2023 Aug 3;15(32):38901–9. doi: 10.1021/acsami.3c05302 (PMC10436242; doi:10.1021/acsami.3c05302)
Supplement: Supplementary file 1 — am3c05302_si_001.pdf [file am3c05302_si_001.pdf]

Supporting Information

Spectral Tuning of a Nanoparticle-on-Mirror System by Graphene Doping and Gap Control with Nitric Acid

*Julia Lawless[1], Oisín McCormack[1], Joshua Pepper[2], Niall McEvoy[2], A. Louise Bradley[1]**

1. School of Physics and AMBER, Trinity College Dublin, College Green, Dublin 2, Ireland

2. School of Chemistry and AMBER, Trinity College Dublin, College Green, Dublin 2, Ireland

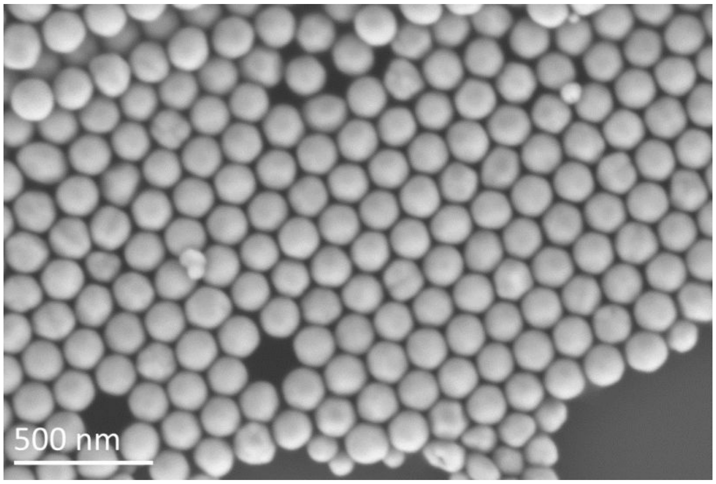

Figure S1: A SEM image of a typical sample of synthesized Au nanospheres. They are uniform in both their size and in their spherical shape, as is evidenced in their natural hexagonal packing after being drop-cast onto the substrate.

*Corresponding author

Email address: bradlel@tcd.ie (A. Louise Bradley)

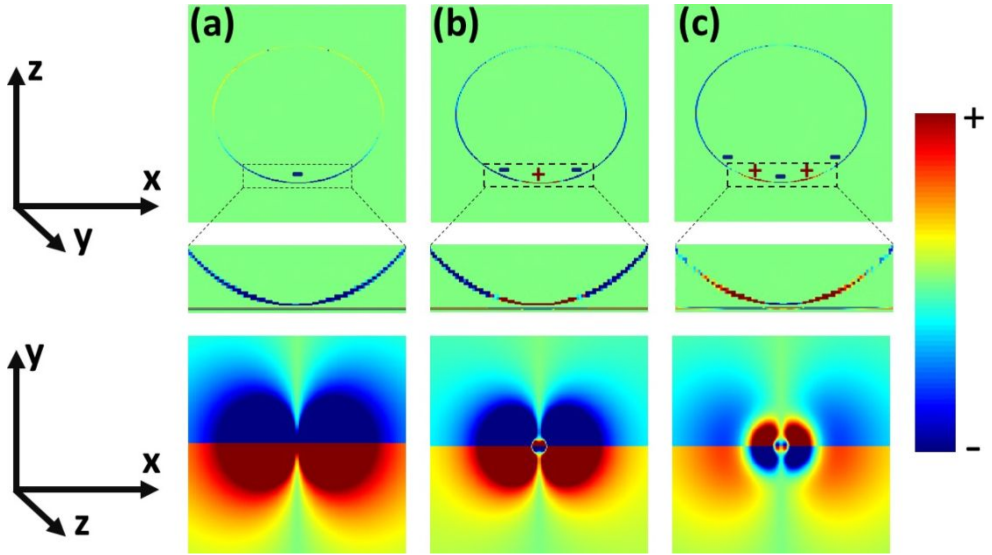

Figure S2: Charge distribution maps from simulations as in figure 2(i)-(iii) in the main text, but replacing the polymer layer with an air layer. The top panel shows the charge distribution through the center of the nanosphere and the substrate, with a closer map shown below giving better resolution to the charge at the top of the Au film and the graphene layer. The bottom panel shows the charge distribution at the top of the Au film, directly underneath the Au sphere. Each map shows a cross setion of 200 nm 200 nm, with the closer-up versions of the top panel maps showing 100 nm 23 nm. The maps show the charge distribution for the (a) dipolar, (b) quadrupolar and (c) octupolar modes. These maps give a simplified approximation to figure 2(i)-(iii) in the main text, as in this case the charge in the sphere does not leak out into the polymer layer.

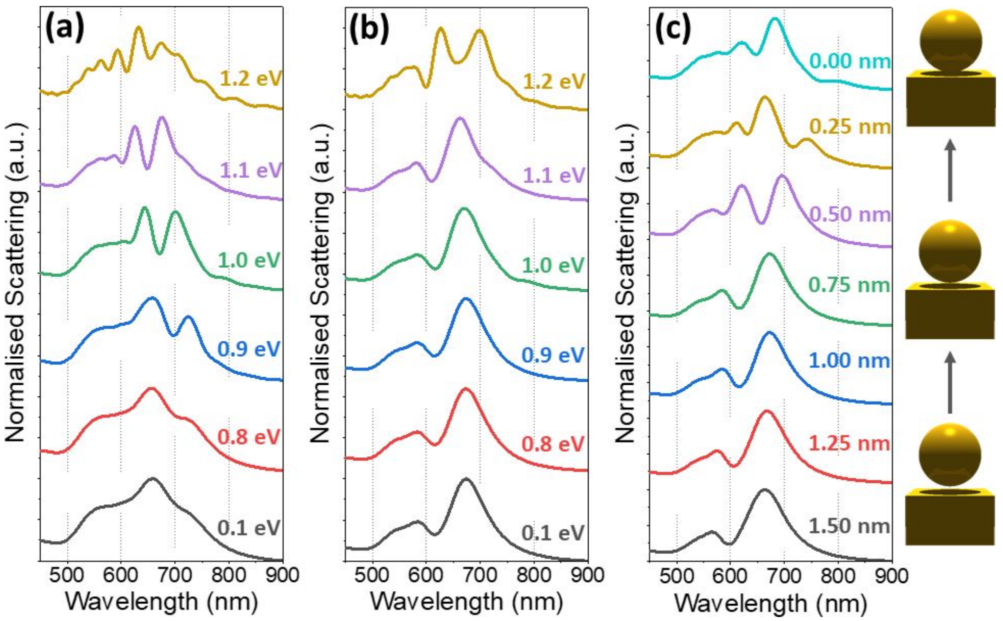

Figure S3: (a,b) Simulated scattering spectra for a 150 nm Au sphere on a 100 nm Au film, sandwiching a monolayer of graphene on a SiO_2/Si substrate with a 0.25 nm and a 1 nm gap between the sphere and the graphene, respectively. The chemical potential of the graphene is increased from 0.1 eV to 1.2 eV. (c) Simulated scattering spectra for the same system, without the graphene layer. The gap between the Au film and Au sphere is reduced from 1.5 nm to 0 nm. This effect is illustrated to the right. These simulations are the same as in figures 2a-c in the main text, but with the gap between the sphere and substrate modelled as air instead of PMMA.

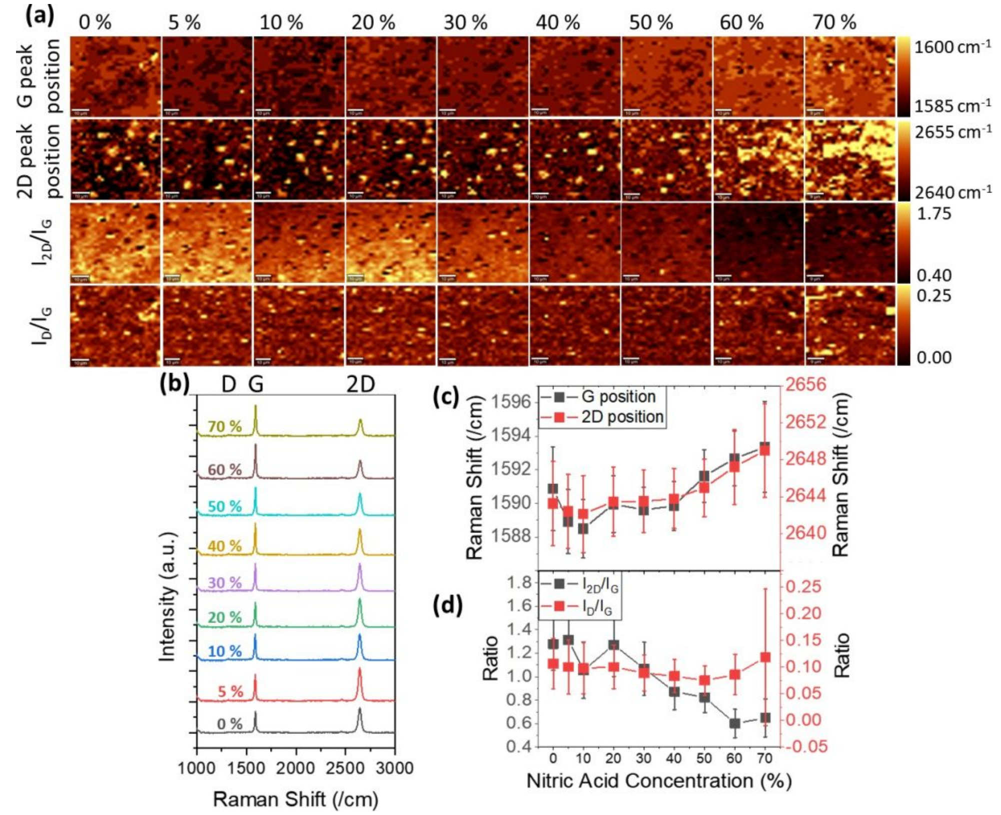

Figure S4: (a) Raman maps of the same area within a graphene sample, showing the position of the G peak and the 2D peak and the intensity of the 2D peak and the D peak after each immersion in nitric acid from 0% to 70%. (b) Raman spectra averaged over each map shown in (a). (c) Positions of G peak and 2D peak taken from (a). (d) Ratio of the intensities of 2D peak and D peak to the G peak taken from (a). The data points show the mean value, and the error bars show the standard deviation. The blue-shifting of the G peak and the 2D peak and the reduced intensity of the 2D peak indicate a high level of doping. The D peak has a very low intensity for all spectra, indicating the high quality of the graphene.

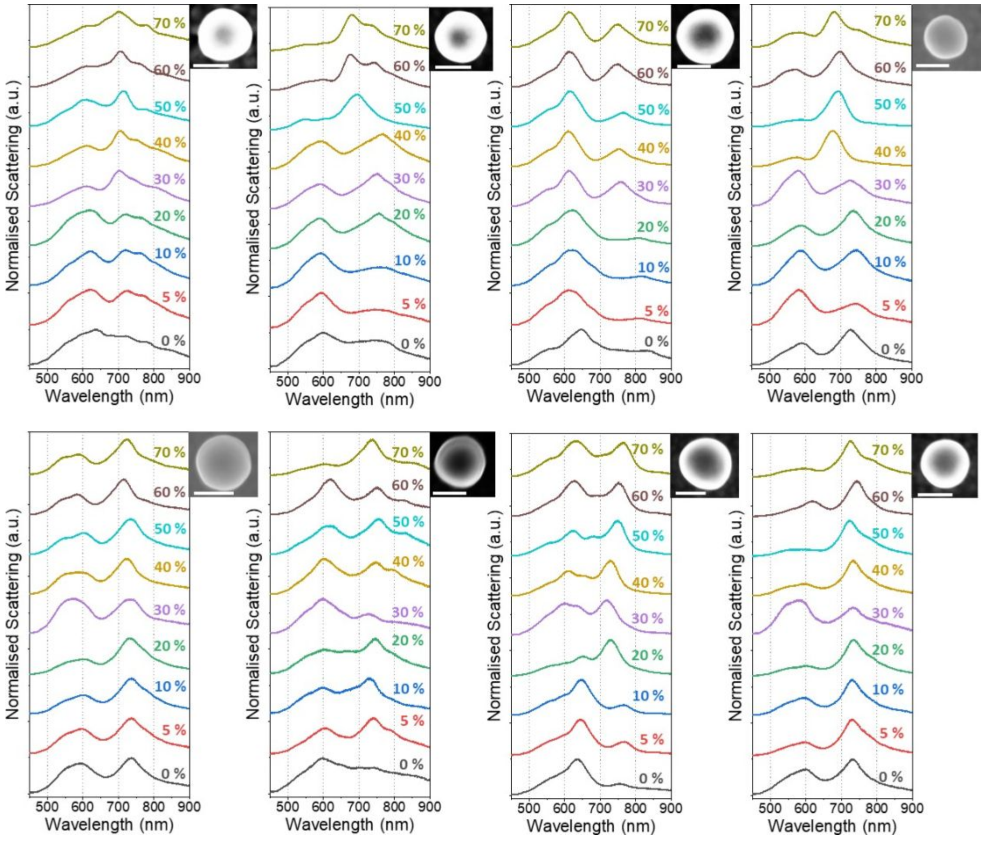

Figure S5: Experimental spectra of 150 nm Au spheres on a 100 nm Au film with an intermediate layer of graphene. Spectra are taken after immersing the sample in 0-70% nitric acid, as in figure 4a in the main text. Corresponding SEM images are shown to the right of each spectrum. Scale bars show 100 nm. The scattering peaks are shown to blue-shift due to the doping of the graphene layer (especially the dipolar mode), before red-shifting again as the gap size decreases. The dipolar mode is shown to split into two peaks.

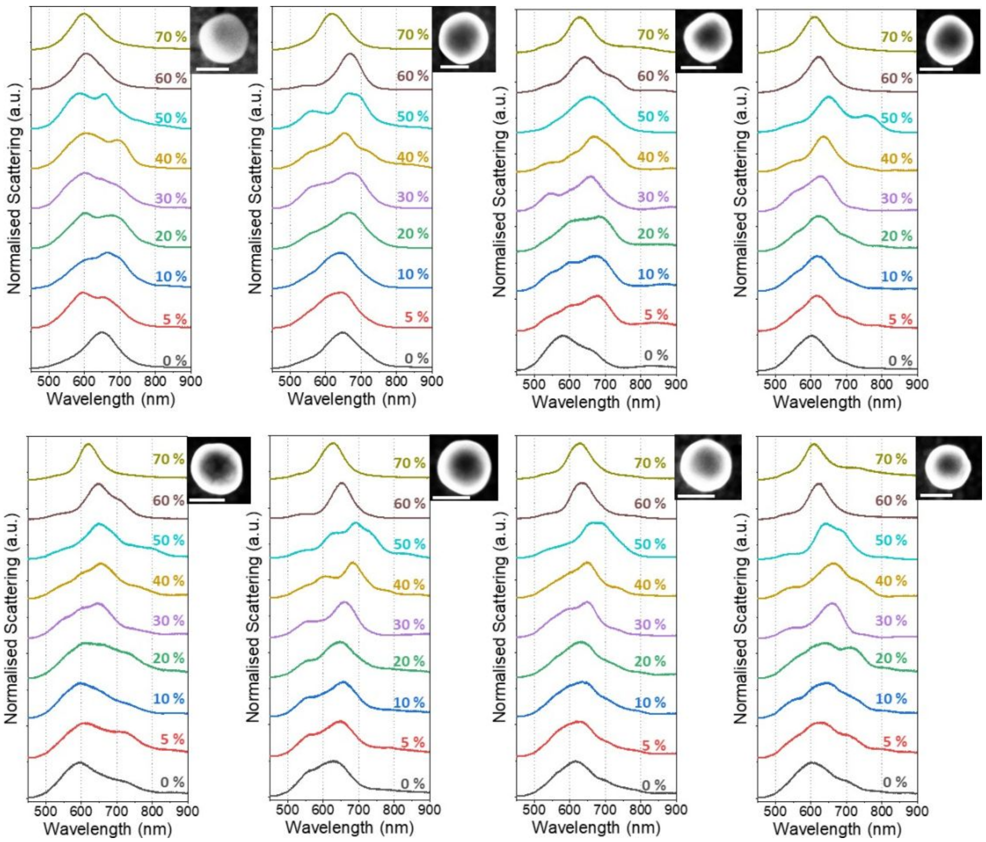

Figure S6: Experimental spectra of 150 nm Au spheres placed directly on a 100 nm Au film, with no intermediate layer of graphene. Spectra are taken after immersing the sample in 0-70% nitric acid, as in figure 3c in the main text. Corresponding SEM images are shown to the right of each spectrum. Scale bars show 100 nm. The peaks are shown to red-shift as the gap decreases before blue-shifting again, collapsing into a single, blue-shifted peak as the neck between the nanosphere and film forms.

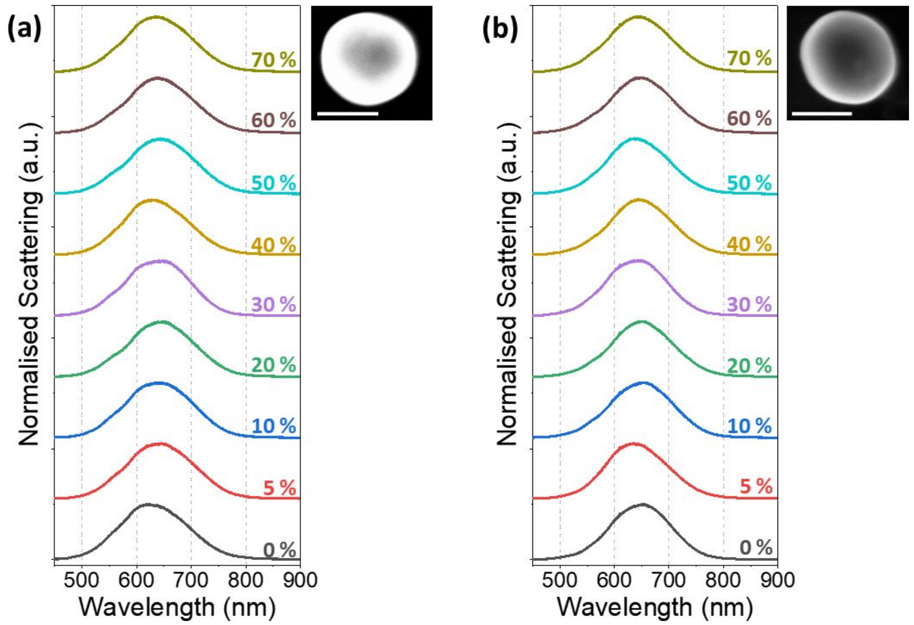

Figure S7: (a) Scattering spectra of a 150 nm Au nanosphere on a monolayer of graphene on a SiO_2/Si substrate. Spectra were taken of the same particle after it was immersed in nitric acid of concentrations up to 70%. The corresponding SEM image is shown to the right, with a white scale bar indicating 100 nm. (b) Scattering spectra of a 150 nm Au nanosphere directly on a SiO_2/Si substrate. Spectra were taken of the same particle after it was immersed in nitric acid of concentrations up to 70%. The corresponding SEM image is shown to the right, with a white scale bar indicating 100 nm. Both sets of data indicate that very little change occurs to the plasmon energy of this system without the presence of the Au film underneath the nanosphere to increase the electric field strength within the gap.
